# Supplementary material for: Phenol-Enriched Virgin Olive Oil Promotes Macrophage-Specific Reverse Cholesterol Transport In Vivo
Source: Biomedicines. 2020 Aug 3;8(8):266. doi: 10.3390/biomedicines8080266 (PMC7460104; doi:10.3390/biomedicines8080266)
Supplement: Supplementary file 1 [file biomedicines-08-00266-s001.pdf]

biomedicines

MDPI

Table S1. Daily ingested dose of hydroxytyrosol and its derivatives through refined olive oil (ROO), functional virgin olive oil (FVOO), and phenolic extract (PE).

Phenol (mg/kg body weight)	ROO	FVOO	PE
Hydroxytyrosol	0.01	0.06	0.14
3,4-DHPEA-AC	0.00	0.23	0.03
3,4-DHPEA-EDA	0.01	1.85	2.29
3,4-DHPEA-EA	0.03	0.20	0.26
**Total hydroxytyrosol derivates**	**0.05**	**2.33**	**2.71**

3,4-DHPEA-AC: 3,4-dihydroxyphenyl ethyl acetate (hydroxytyrosol acetate); 3,4-DHPEA-EA: 3,4-dihydroxyphenylethanol-elenolic acid (oleuropein aglycone); 3,4-DHPEA-EDA: 3,4-dihydroxyphenylethanol-elenolic acid dialdehyde (dialdehydic form of elenolic acid linked to hydroxytyrosol). Values are expressed as mg/kg body weight.

Table S2. Fatty acid compositions of refined olive oil (ROO) and functional virgin olive oil (FVOO).

Fatty acids (%)		ROO	FVOO
Caprylic acid	C8:0	0.00	0.00
Capric acid	C10:0	0.00	0.00
Lauric acid	C12:0	0.00	0.00
Myristic acid	C14:0	0.00	0.00
Palmitic acid	C16:0	11.96	11.96
Margaric acid	C17:0	0.06	0.06
Stearic acid	C18:0	3.08	3.08
Arachidic acid	C20:0	0.41	0.41
Behenic acid	C22:0	0.11	0.11
Lignoceric acid	C24:0	0.05	0.05
**Total saturated**		**15.67**	**15.67**
Palmitoleic acid	c 9-C16:1	1.00	1.00
Oleic acid	c 9-C18:1	72.71	72.71
Gadoleic acid	c 11-C20:1	0.25	0.25
**Total monounsaturated**		**73.96**	**73.96**
Linoleic acid	c 9,12-C18:2	9.39	9.39
Timnodonic acid	C18:5 n3	0.20	0.20
Linolenic acid	c 9,12,15-C18:3 n3	0.54	0.54
Eicosatetraenoic acid	c 8,11,14-C20:3 n6	0.00	0.00
**Total polyunsaturated**		**10.13**	**10.13**

cc BY

© 2020 by the authors. Submitted for possible open access publication under the terms and conditions of the Creative Commons Attribution (CC BY) license (http://creativecommons.org/licenses/by/4.0/).
